# Fluconazole-Resistant Vulvovaginal Candidosis: An Update on Current Management

**DOI:** 10.3390/pharmaceutics16121555

**Published:** 2024-12-04

**Authors:** Karolina Akinosoglou, Achilleas Livieratos, Konstantinos Asimos, Francesca Donders, Gilbert G. G. Donders

**Affiliations:** 1 Department of Medicine, University of Patras, 265 04 Rio, Greece; 2 Department of Internal Medicine and Infectious Diseases, University General Hospital of Patras, 265 04 Rio, Greece; 3 Independent Researcher, 152 38 Athens, Greece; 4 Metropolitan General, Leof. Mesogeion 264, 155 62 Athens, Greece; asimos.kon@gmail.com; 5 Femicare, Clinical Research for Women, 3300 Tienen, Belgium; francesca.donders@gmail.com (F.D.); gilbert.donders@gmail.com (G.G.G.D.); 6 Regional Hospital Heilig Hart, 3300 Tienen, Belgium; 7 Department of Obstetrics and Gynecology, University Hospital Antwerpen, 2650 Antwerp, Belgium

**Keywords:** fluconazole-resistant vulvovaginal candidosis, fluconazole-refractory vulvovaginal candidosis, recurrent vulvovaginal candidosis

## Abstract

Currently, the rising prevalence of resistant *Candida* species, particularly *Candida albicans*, as well as non-albicans isolates such as *Candida glabrata* and *Candida krusei*, represent challenges in their management. In this review, we aimed to explore the current management of fluconazole-resistant vulvovaginal candidiasis (FRVVC). Identified studies focused on alternative antifungal therapies, including boric acid, nystatin, and newer agents like oteseconazole and ibrexafungerp. The findings highlight the need for tailored treatment regimens, considering the variability in resistance patterns across regions. Unprofessional as well as professional overuse of antifungals for vulvovaginal symptoms that are not caused by *Candida* infections should be combatted and banned as much as possible. Instead of high-dose maintenance regimens using weekly doses of 150 to 200 mg of fluconazole for 6 months or longer, it is advisable to use an individualised degressive regimen (ReCiDiF regimen) in order to tailor the treatment of a particular patient to the lowest dosage possible to keep the diseases controlled. Additionally, this report underscores the impact of antibiotic use on the microbiota, which can raise the possibility of VVC and lead to fluconazole resistance, emphasizing the necessity for cautious antibiotic prescribing practices.

## 1. Introduction

Vulvovaginal candidiasis (VVC) is a commonly encountered disease that affects about three out of four women during their lifetime [1,2,3]. A subset of these patients has recurrent episodes of this condition termed recurrent vulvovaginal candidiasis (RVVC). The condition is diagnosed by the presentation of a minimum of three to four episodes of VVC in a single year, impacting approximately 5–8% of women in their reproductive years [1,2,3]. The primary pathogen responsible for VVC is *Candida albicans*, which accounts for more than 90% of cases, although non-albicans *Candida* (NAC) species are increasingly recognized as contributors to these infections [1,2,3,4,5]. Some contraceptives, pregnancy, use of poorly ventilated clothing, HIV infection, immunosuppressive agents, diabetes mellitus, antibiotic use, atopic disease, and genetic predisposition are common factors that promote RVVC [6,7]. According to some, even bacterial vaginosis (BV), oral sex, and the use of soap are predisposing factors, but the list is long and the evidence is scarce [8,9].

Among antifungal treatments, fluconazole is commonly utilized due to its effectiveness and ease of use. Nevertheless, the rising frequency of fluconazole-resistant *C. albicans* (FRCA) presents a growing obstacle to successful treatment [5,10,11,12]. Historically, fluconazole resistance in *C. albicans* was deemed uncommon, representing less than 5% of isolates with RVVC [13,14]; however, there has been a notable increase in recent years [15,16]. Several mechanisms have been implicated in fluconazole-resistant VVC (FRVVC), including altered ergosterol synthesis, altered sterol and azole import, the overexpression of membrane transporters, as well as genomic and chromosomic variations reducing susceptibility to therapy [17].

Research indicates a growing incidence of FRCA among women with RVVC, particularly those with significant prior exposure to fluconazole [1,2,3]. This increase is likely linked to the extensive usage of fluconazole for both acute and maintenance treatment of VVC, which exerts selective pressure favouring resistant strains [1,2,3]. The median minimum inhibitory concentration (MIC) of fluconazole for resistant isolates varies considerably, often requiring the consideration of alternative therapeutic approaches [1,2,3,4,5].

The inherent fluconazole resistance among NAC species has also become a growing issue, especially as these species are being increasingly identified in VVC cases [10,11,15,16,18,19]. *C. albicans* was traditionally recognized as the primary cause of VVC; NAC species were once considered responsible for a smaller fraction of cases [20,21,22]. In recent years, however, clinicians have encountered a notable prevalence of NAC isolates, such as *C. glabrata* (Nakaseomyces glabrata) and *C. krusei* (Pichia kudriavzevii) [23], in some particular areas, with some studies indicating their involvement in up to half of RVVC cases [7,24,25]. Similarly to FRCA, the increasing prevalence of fluconazole-resistant NAC species is closely linked to the widespread use of fluconazole [7,24,25]. This extensive use has contributed to fluconazole resistance, particularly for *C. glabrata* and *C. krusei*, which are inherently less susceptible to azoles or exhibit cross-resistance to other azole drugs [7,24,25].

This resistance complicates treatment strategies, as these species often require alternative antifungal therapies that are less commonly used and may have more side effects. Managing VVC that is resistant to fluconazole necessitates the use of alternative antifungal treatments, given the limited effectiveness of standard therapies [7,24,25]. Treatment options include (a) boric acid, which may accomplish high clinical and mycological cure rates and offers symptomatic relief as well as helps reduce recurrence rates; (b) nystatin, which may serve as a viable alternative, particularly for NAC infections; (c) a switch to vaginal capsules or creams containing antifungal agents, sometimes in combination, which can provide targeted treatment with more favourable safety profiles than oral formulations; (d) topical antifungals like itraconazole and ketoconazole, which require careful monitoring for hepatic toxicity and other adverse effects when used orally, thereby rendering them less suitable for prolonged use; (e) augmentation of local fungicidal effect with potentiators like ibuprofen or domiphen bromide, (f) newer agents, such as oteseconazole or ibrexafungerp, which may provide more effective management and (g) combination therapies; e.g., an azole combined with a non-azole therapy [1,2,3,4,25,26,27,28].

We hereby expand on previous findings and guidelines regarding the management of VVC, particularly fluconazole-resistant infections [7]. This study, therefore, seeks to investigate the recent literature to evaluate treatment options and clinical outcomes for patients with FRVVC. Finally, we end with discussing some essential preventive measures to dam the antifungal resistance epidemic.

## 2. Materials and Methods

**Figure 1:**
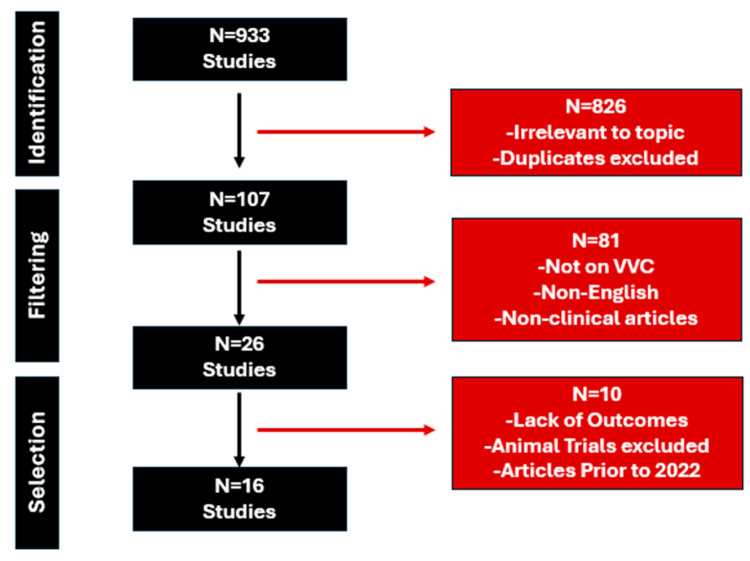
Diagram of included studies.

We performed extensive searches in PubMed, Medline, and Cochrane databases from 1 January 2000 to 1 September 2024. The search strategy included the following parameters: Vulvovaginal Candidosis OR Vulvovaginal Candidiasis OR Fluconazole Resistant OR Recurrent Vulvovaginal Candidiasis OR Fluconazole Refractory AND Therapy AND Outcomes. A total of 933 studies were selected for further examination. After a closer examination of these 933 articles, 826 were excluded as they were out of scope (unrelated topic or duplicates). Only English-language studies were examined further. Moreover, 26 articles were reviewed for final-stage selection after 81 studies were excluded due to language criteria or work unrelated to VVC or non-clinical articles. Ultimately, 16 articles on the management of fluconazole-resistant clinical cases were specifically included in this work. Only articles since 2002 were included to account for the evolution of clinical practice when managing recurrent VVC. Three independent researchers participated in this selection process. An illustration of included studies is visualized in Figure 1.

## 3. Results

Fluconazole resistance was defined in all studies through in vitro analysis. The overall most widely used methodology was in vitro antifungal susceptibility testing of different variations (n = 10). A representative sample (n = 8) of recent epidemiological studies on the frequency of fluconazole-resistant VVC is presented in Table 1. As fluconazole resistance would vary significantly for older publications, only articles published in the last decade were included from different countries/regions to better capture the global epidemiological footprint of fluconazole resistance in VVC patients. Data from prevalence studies highlight the variability in fluconazole resistance among different *Candida* species and across various regions. *C. albicans* was the most prevalent species in all the regions studied, with its prevalence ranging from 32.4% in China to 88.2% in Iran (Table 1). Resistance rates for *C. albicans* vary significantly, with the lowest being approximately 2% in Ethiopia and the UK and no resistance detected in Iran [26,27]. However, in the USA, *C. albicans* showed a resistance rate of 23% at a neutral pH of 7.0, which increased to 52% at a lower pH of 4.5, indicating that conditions significantly influence resistance (Table 1).

*C. glabrata* was the second most common species in several regions, with a notable prevalence of 43% in Turkey, 13.6% in Greece, and 6.8% in the UK (Table 1). *C. krusei* consistently showed high resistance across the studies, with a 100% resistance rate reported in both Turkey and Ethiopia [26,29]. The data underscore the importance of regional surveillance, as resistance patterns differ, necessitating tailored antifungal strategies depending on the specific *Candida* species and local resistance profiles.

**Table 1:** Representative studies on the prevalence of fluconazole-resistant VVC.

Author	Year	Origin	Study Design	*Candida* Species	Diagnosis Method	Prevalence of Resistance
J. D. Sobel [30]	2023	USA	Longitudinal, Observational study over a 10-year period	*C. albicans* was the dominant species (76.3% of positive yeast isolates)	Antibiotic susceptibility tests in line with guidelines MICs for pH 7.0 and 4.5	pH 7.0: 23% of isolates were resistant (MIC ≥ 8 mg/mL) pH 4.5: resistance rates were 52% of the isolates
S. Maraki [31]	2019	Greece	6-year Observational study	*C. albicans* (75.6%) *C. glabrata* (13.6%)	Isolation on Sabouraud dextrose agar, identification using VITEK card.	Overall resistance rates: 6.6% to fluconazole
D.N. Anh [32]	2021	Vietnam	Cross-sectional study	*C. albicans* (51.37%) *C. parapsilosis* (25.88%) *C. glabrata* (11.37%) *C.tropicalis* (4.31%) *C. krusei* (3.92%) *C. africana* (1.57%) *S. cerevisiae* (0.78%) *C. nivariensis* (0.39%) *C. lusitaniae* (0.39%)	Direct microscopic examination (10% KOH) Species identification was performed using morphological tests, PCR, and sequencing	Resistance rate to fluconazole was 4.35%
W. Wang [19]	2024	China	Retrospective Observational study	*C. albicans* (32.40%) *C. tropicalis* (17.80%) *C. glabrata* (13.70%) *C. parapsilosis* (8.63%)	Antifungal susceptibility testing using ATB^®^ FUNGUS 3.	*C.albicans* exhibited a fluconazole resistance rate of 5.2%. *C. tropicalis* showed significant resistance to fluconazole of 38.3%
A. Bitew and Y. Abebaw [26]	2018	Ethiopia	Cross-sectional study	*C. albicans* (58.6%) *C. krusei* (17.2%) *C. dubliniensis* (9.2%) Other included *C. glabrata*, *C. tropicalis*	Susceptibility testing using VITEK system	Highest resistance was observed against fluconazole (17.2% overall). *C. krusei*: 100% resistance to fluconazole. *C. albicans*: 2% resistance rate to both fluconazole
A. Rezaei-Matehkolae [27]	2016	Iran	Cross-sectional study	*C. albicans* (88.2%) *C. glabrata* (8.8%) *C. kefyr* (2.9%)	Classical mycological tests, PCR-RFLP method for molecular identification	Resistance was not detected among the isolates for fluconazole
F.G. Hösükoğlu [29]	2022	Turkey	Observational study	*C. albicans* (47%) *C. glabrata* (43%) *C. kefyr* (5%) *C. krusei* (2%) *C. tropicalis* (2%) *C. guilliermondii* (1%)	Antifungal susceptibility of these isolates using the reference broth microdilution method as per CLSI guidelines	*C. albicans*: 21.3% resistant *C. krusei*: 100% resistant (intrinsic resistance)
Ratner JC [33]	2024	UK	Retrospective Observational study	*C. albicans*: 87.4% *N. glabrata*: 6.8% *P. kudriavzevii*: 0.55% *C. dubliniensis*: 1.64% *Meyerozyma guilliermondii*: 0.27% *Clavispora lusitaniae*: 0.82% *C. parapsilosis*: 2.19% *C.tropicalis*: 0.27%	Fluconazole resistance assessed using the disc diffusion method and Sensititre YeastOne assay as per CLSI	Resistant species *C. albicans*: 2020–21: 2.19% *Nakaseomyces glabrata [Candida glabrata]*: 2020–21: 0.27% *Pichia kudriavzevii [Candida krusei]:* 2020–21: 0.55%

The included studies on the management of fluconazole resistance in *Candida* infections reveal a comprehensive investigation into various *Candida* species, with *C.albicans* being included in all studies but 1, while the most identified NAC species like *C.glabrata* was present in 16 studies (Table 2). Resistance was predominantly determined through in vitro antifungal susceptibility testing (six studies), broth microdilution (three studies), E-test methods (one study), clinical symptoms (two studies), and the use of a Candifast kit (one study) [1,2,18,19,20,21,22,24,33,34,35,36,37,38,39,40]. Notably, all studies but one included a fluconazole arm in their study design. Following resistance diagnosis, treatments varied widely, including the use of boric acid, ketoconazole, itraconazole, otesoconazole, voriconazole, nystatin, amphotericin B, flucytosine, and ibrexafungerp.

When ranking the effectiveness of various treatments for fluconazole-resistant *Candida* infections, O-oteseconazole stands out as the most effective, with a weighted overall success rate of 88% and a 71% clinical cure rate [19,38]. Boric acid follows with a weighted success rate of 77%, making it a strong treatment option for fluconazole-resistant cases [2,18,34,36,37,40,41]. Voriconazole comes next with a 73% clinical cure rate [7]. Ibrexafungerp also shows effectiveness, with a 70% clinical cure rate by day 25 [34]. Finally, nystatin offers a mycological cure rate of 56% in fluconazole-resistant cases [1]. The worst-performing studies in terms of treatment failures include a broad range of *Candida* species, particularly NAC species like *C. glabrata* and *C. krusei*, which are known for their resistance to common antifungal treatments [7,25]. Richter SS et al. had the highest failure rate at 51.4%, particularly struggling with *C. glabrata* [22]. Fan S et al. further illustrate the treatment difficulties of fluconazole-resistant isolates, where nearly half of the cases failed to respond to nystatin [1]. These studies underscore the necessity of utilizing multiple antifungal agents and tailored treatment regimens to effectively manage resistant cases.

## 4. Discussion

We aimed to explore the current literature pertaining to FRVVC and its management. Data from prevalence studies highlighted the variability in fluconazole resistance among different *Candida* species and across various regions. Following resistance diagnosis, treatments varied widely, including the use of boric acid, ketoconazole, itraconazole, otesoconazole, voriconazole, nystatin, amphotericin B, flucytosine, and ibrexafungerp. Otesoconazole and boric acid proved to be the more effective, followed by voriconazole, ibrexafungerp, and nystatin.

In cases of fluconazole-resistant VVC (FRVVC), alternative treatment plans, such as a degressive dosing regimen, are currently being explored [7,10,25]. This approach starts with a standard fluconazole dose and gradually reduces the frequency of administration over time [7,10,25]. The objective is to control *Candida* overgrowth and prevent recurrences by maintaining sufficient antifungal levels while minimizing the risk of drug resistance and side effects from long-term, high-dose therapy [7,10,25]. This strategy aims to manage and prevent recurrent infections while reducing the overall antifungal load. The approach to managing RVVC after a maintenance regimen depends on the frequency of recurrences. If recurrences are infrequent, each episode can be treated on a case-by-case basis. However, if frequent recurrences return, it is advised to reinitiate, extend, or modify the induction and maintenance regimens to better control the condition [7,25,34]. In clinical settings, experts have noted that treatment durations typically may last up to half a year, although approximately 60% of women relapse after discontinuing maintenance treatment [14,42,43]. In more challenging cases, maintenance treatment may extend beyond 6 months, sometimes continuing for years, which is not unusual in clinical practice for severe RVVC cases [37,38,41]. Within the ReCiDiF regimen, the dosing schedule is progressively reduced as symptoms come under control, with the regimen usually lasting for a year for patients who respond well. These patients can stop treatment after this period [10,14,42,43]. For those considered “suboptimal responders,” the regimen may require further adjustment and an extended duration [10,14,42,43]. In some cases, patients may need to continue taking one tablet per month or every two weeks, depending on how frequently they relapse and how well they respond during the treatment course [10,14,42,43]. As a primary measure, there is a proper diagnosis prior to treatment that determines whether this is professionally installed or driven by peer or pharmacist advice without examination, which is imperative in order to diminish the improper and frequent use of antifungals for reasons that may not be *Candida* infection. It is known that patients very often misdiagnose themselves based on their assumption of what causes their vulvovaginal discomfort [44].

Topical treatments, including vaginal creams with azole antifungals, are frequently used in managing VVC, especially FRVVC [7,10,25]. These creams deliver medication directly to the infection site, achieving higher local drug concentrations while minimizing systemic side effects [7,10,25]. This approach is especially beneficial in cases where oral antifungals, such as fluconazole, have failed due to resistance [7,10,25]. Clotrimazole, miconazole, terconazole, and boric acid are recommended for recurrent VVC caused by both *C. albicans* and NAC species [45]. Boric acid has shown high cure rates and low short-term recurrence rates in FRCA infections [2]. Topical therapy, except for boric acid due to teratogenic effects [46], is preferred for pregnant women, breastfeeding mothers, and those with potential drug interactions or previous adverse effects from oral azoles [47]. The prolonged contact with the affected area enhances the potential for eradicating resistant strains, particularly when combined with additional therapies like boric acid or probiotics [7,10,25]. This multi-faceted treatment aims to counter various risk factors for recurrence, including the intestinal *Candida* reservoir, mycotic biorhythm, biofilm formation, and the presence of non-*albicans* species or *G. vaginalis* [48]. The use of topical antifungals can also be potentiated by the addition of adjuvant substances, such as domiphen bromide or low doses of ibuprofen [28,49,50] (ref). In humans, a combination of different doses of domiphen bromide with miconazole in a vaginal cream was recently tested in a phase 1 trial by our group, resulting in promising amelioration of the latter’s effect on the candida vulvovaginitis, as compared with miconazole cream with the additional domiphen bromide.

Probiotics have gained attention as a complementary treatment aimed at restoring and maintaining a balanced vaginal microbiome [7,25]. In particular, those containing *Lactobacillus* species have been investigated as both a preventive and adjunctive treatment for VVC [7,10,25]. These beneficial bacteria help sustain an acidic vaginal environment and compete with *Candida* species, potentially lowering the infection risk [7,10,25]. Although evidence from randomized controlled trials is still evolving, the use of probiotics alongside antifungal therapy has shown the potential to decrease the recurrence of VVC by strengthening the vaginal ecosystem’s natural defences [7,25]. However, current evidence is not yet robust enough to be universally included in clinical guidelines, and additional research is necessary to better define the role of probiotics in preventing and treating FRVVC [7,10,25,51].

The use of systemic antibiotics has been associated with disruptions in the vaginal microbiome, increasing the risk of VVC, particularly in women who experience recurrent infections [7,10,25]. Moreover, we are now aware of the rising levels of azole antifungal resistance, as shown by in vitro testing of *Candida* in women with complicated RVVC [33]. However, we need more information on how this affects patient management, as treating these infections is challenging. The exact causes of this increase are still uncertain, but it appears to be linked to a policy shift that promoted the empirical treatment of VVC based on symptoms and signs or over-the-counter drugs rather than prescriptions following wet mount microscopy or yeast cultures or seeking medical care. Empirical treatment of VVC should be discouraged to prevent further growth of FRCA and fluconazole-resistant NAC species. The clinical management of fluconazole-resistant VVC encompasses not only the identification of suitable antifungal therapies but also the education of patients and their adherence to treatment protocols [31,32,52]. Regular follow-up is crucial for assessing treatment efficacy and addressing potential side effects. Furthermore, healthcare providers should stress the importance of avoiding unnecessary antifungal use to mitigate the risk of developing resistance [29,34,35,53].

In the era of rising antifungal resistance, with an increased need for safe regimens with minor adverse effects, minimal drug–drug interactions, and longer half-times that allow less frequent administration and outpatient care, new agents in currently used classes of antifungals and new compounds with novel mechanisms of action have now been developed. A number of those have already been approved by the European Medical Agency and the U.S. Food and Drug Administration for the treatment of VVC, including oteseconazole and ibrexafungerp. In phase III studies by Martens et al. and Sobel/Donders et al., oteseconazole showed very promising results [38,54], leading to a recurrence rate as low as 4 to 5% in cases of FRVVC. Also, in comparison with fluconazole, clinical and mycological cure rates were higher in the oteseconazole arms (71.3 and 82.5% with oteseconazole versus 56.0 and 59.1 in the fluconazole arm, respectively) [19]. In the same context, the need for rescue medication and clinical cure rates on day 25 favored ibrexafungerp in a recent randomized clinical trial by Nyirjesy et al. [34].

It would be interesting to explore how other new agents, now tested for invasive fungal infection, would perform in mucocutaneous disease, including VVC. Fosmanogepix is a novel Gwt1 inhibitor [55] that blocks inositol acylation during GPI-anchored protein biosynthesis and offers an oral availability of over 90% beyond its intravenous formulation. It is active against many pathogenic molds and yeast, including most Candida species, including strains resistant to echinocandins and triazoles [56]. However, *Candida krusei* and related species exhibit intrinsic resistance to fosmanogepix [57]. In phase II trials for candidemia, fosmanogepix was shown to be safe and well-tolerated, achieving an 80% treatment success rate and an 85% survival rate at day 30 [58,59]. Currently, a phase III clinical trial is evaluating the efficacy and safety of fosmanogepix for candidemia and invasive candidiasis. Rezafungin is a new long-acting echinocandin, enabling once-weekly intravenous administration with front-loaded dosing. It has shown in vitro efficacy against several *Candida* species while two recent trials, a phase II (STRIVE) and a phase III (ReSTORE) study, compared rezafungin to caspofungin for treating candidemia and invasive candidiasis [60,61,62,63]. Rezafungin proved non-inferior to caspofungin, meeting the EMA’s global cure endpoint on day 14 and the FDA’s all-cause mortality endpoint on day 30, leading to its approval for invasive candidiasis [60]. Of note, its efficacy remained consistent across various *Candida* species, including fluconazole-resistant and FKS mutant strains, regardless of MIC values [64]. Lastly, ATI-2307 (or T-2307) is an investigational antifungal agent classified as an aromatic diamidine. It works by inhibiting respiratory chain complexes III and IV, disrupting the mitochondrial membrane potential in yeast cells [65]. This drug shows broad-spectrum activity, targeting *Candida* species, including strains resistant to echinocandins and azoles [66,67]. Though still in the early stages of development or study, so far, available data on all these regimens have shown promising outcomes, so one can wonder whether their use can expand in FRVVC.

### Limitations

Our study suffers a number of limitations. Although FRCA or fluconazole-resistant NAC isolates were noted in the studies explored, it remains unknown whether fungi were acting as colonizers or actual pathogens. In this context, *C. glabrata* has been identified as a non-pathogenic saprophyte, especially among people living with HIV or diabetes and those who are immunocompromised [68]. In the majority of studies, no data are provided regarding microscopic examination that could identify the presence of pseudomycelia; hence, the presence of an infection was recorded based on culture data only. Similarly, no data on repeated or confirmative testing or compliance with guidelines [42,69,70] on the diagnosis of RVVC was noted, leading to overdiagnosis or misinterpretation. Also, in the studies where MIC was measured, we have to keep in mind that fluconazole resistance through in vitro susceptibility testing does not always correlate with phenotypic resistance, unlike in antibiotic resistance testing of bacteria. The trailing growth phenomenon that *Candida* exhibits due to the diversity of resistance mechanisms and its ability of biofilm formation prevents directly translating in vitro susceptibility to in vivo efficacy [71,72]. On the other hand, negative cultures may not always reflect active disease but rather recent treatment, bad swab quality, the presence of growth inhibitors, or missed sample location. Although our review spans the last 25 years of practice, the non-inclusion of English-language papers and databases other than PubMed, Medline, and Cochrane may have led to important experiences being overlooked. Lastly, at the time of the publication of this review, it is possible that the rapidly evolving field of antifungal therapeutics has developed more regimens than discussed herein [73].

## 5. Conclusions

The management of VVC, particularly in women prone to frequent relapses, remains a significant challenge despite the efficacy of current antifungal treatments like fluconazole and itraconazole. While these therapies can alleviate acute infections, a substantial proportion of patients experience only temporary symptom relief, necessitating long-term maintenance strategies to prevent recurrent episodes. However, even with carefully monitored regimens, a significant number of women continue to suffer from multiple recurrences, highlighting the limitations of existing approaches.

The rising prevalence of FRVVC further complicates this issue, underscoring the urgent need for alternative therapeutic strategies and robust antifungal stewardship. Clinicians must adopt a comprehensive approach that balances effective treatment with efforts to reduce the emergence of antifungal resistance. This includes prioritizing topical therapies whenever feasible, optimizing treatment regimens to preserve the natural vaginal flora, and minimizing the unnecessary use of systemic antifungal medications. A healthy vaginal microbiome and restored immune function are crucial in preventing recurrences and maintaining long-term vaginal health.

Emerging treatments, such as newer antifungal agents like oteseconazole and ibrexafungerp, show promise in addressing these challenges and expanding the therapeutic options available to both patients and clinicians. Additionally, the development of a vaccine currently undergoing clinical trials is a highly encouraging step forward, offering the potential for transformative progress in preventing VVC altogether. However, the success of these advancements will depend on appropriate usage guided by well-informed clinical oversight and antifungal stewardship to ensure sustainable outcomes.

Ultimately, addressing the growing issue of recurrent and resistant VVC requires an integrated strategy that combines effective treatment, patient education, systematic monitoring, and continued research into innovative therapies. By adopting a forward-looking and balanced approach, we can improve outcomes for women while safeguarding the effectiveness of antifungal treatments for future generations.

## Figures and Tables

**Table 2:** Representative studies on the management of fluconazole-resistant VVC.

Included Studies	Patients	Inclusion Criteria	*Candida* Species	Intervention	Administration	Study Design	Outcomes
Wang X et al., 2024 (China) [19]	322 10% Resistant in Fluconazole arm 2% Resistant in Oteseconazole arm	18 to 75 years VSS score ≥ 7 Resistance determined through in vitro antifungal susceptibility testing	*C. albicans* *C. glabrata* *C. tropicalis* *C. krusei* *C. spherical* *C. parapsilosis* *K. ohmeri C. dubliniensis* *S. cerevisiae* *C. lusitaniae*	Oteseconazole (oral) Fluconazole (oral)	**Oteseconazole Group**: 600 mg on Day 1, followed by 450 mg on Day 2, Matched placebo on Day 4. **Fluconazole Group**: 150 mg on Day 1, Matching placebo on Day 2, 150 mg on Day 4.	RCT	Mycological cure rate oteseconazole arm 82.50% versus fluconazole arm 59.12% Clinical cure rate oteseconazole arm 71.25% versus fluconazole arm 55.97%
Maftei NM et al., 2023 (Romania) [21]	663 pregnant women *C. albicans*: Fluconazole resistance rate: 8.5% *C. glabrata*: Fluconazole resistance rate: 100% *C. krusei*: Fluconazole resistance rate: 66.7%	Resistance determined using a Candifast kit, which tested various antimycotics, including fluconazole, on isolated *Candida* strains from the patients	*C. albicans* *C. glabrata* *C. krusei*	Econazole Fluconazole Ketoconazole Miconazole Amphotericin B Nystatin Flucytosine	No specific dosages or duration for treatment	Retrospective RWE	Miconazole was effective in over 80% of the strains, making it the preferred treatment for *C. krusei* In Fluconazole-resistant *C. albicans*, miconazole had the highest sensitivity at 93.2%
Marchaim D et al., 2012 (USA) [18]	25 women with fluconazole-resistant recurrent C albicans	Resistance determined using broth microdilution as per CLSI guidelines. Fluconazole resistance defined as MIC of 2 mg/mL or greater.	*C. albicans*	Increased Dosages of Fluconazole (orally) **Boric Acid Vaginal Suppositories** Ketoconazole Itraconazole Gentian Violet Nystatin Suppositories Topical Azoles and Other Antifungals	**Boric Acid**: 600 mg intravaginal suppositories daily for 2 weeks. **Fluconazole**: For low-level resistance (MIC of 2–4 mg/mL), 150–200 mg bi-weekly. **Ketoconazole**: 100 mg every 24 h. **Itraconazole**: 200 mg every 24 h. **Gentian Violet**: Applied daily for 14 days in cases with high-level resistance	Retrospective RWE	**High-Dose Fluconazole**: 11 patients remained asymptomatic with negative vaginal yeast cultures **Boric Acid Vaginal Suppositories**: 3 patients remained asymptomatic **Ketoconazole Therapy**: 4 out of 5 remained asymptomatic and culture-negative **Itraconazole Therapy**: Three patients were successfully managed **Gentian Violet**: 1 patient was treated and remained asymptomatic and culture-negative for over two years
Fan S et al., 2015 (China) [1]	293 Recurrent Vulvovaginal Candidiasis	18 to 50 years old Resistance determined in vitro using the Neo-Sensitabs tablet assay.	*C. albicans* *C. glabrata* *C. tropicalis* *C. parapsilosis* *C. famata*	**Vaginal Nystatin Suppositories** **Oral Fluconazole**	**Nystatin Group**: 20 MU daily vaginal suppositories for 14 days as the induction regimen, followed by 20 MU daily for a week pre-/post- menstruation for half a year as maintenance. **Fluconazole Group**: 150 mg on 1, 4, and 7 days as induction, and then 150 mg every 7 days for half a year as maintenance.	RCT	**Fluconazole-Resistant Cases in the Nystatin Group**: Mycological cure rate in 5/9 cases
File B et al., 2023 (USA) [2]	970 71 (7.3%) with clinically defined fluconazole-resistant *C. albicans*	Resistance defined clinically based on the persistence of symptoms after fluconazole treatment. In vitro susceptibility testing was performed when available, with fluconazole resistance determined by MIC of ≥8 µg/mL.	*C. albicans*	**Prolonged Antifungal Therapy** involved extended courses of oral or topical azole therapy, such as fluconazole **Non-Azole Treatments**: For recurrent or resistant cases, boric acid suppositories were used	Boric Acid: 600 mg intravaginally nightly for at least 14 days. 200 mg of fluconazole taken orally once or twice a week for a duration of 6 months.	Retrospective RWE	Boric acid: The mycological and clinical cure rates were 85.7% and 73.7%, respectively.
Morris GC et al., 2022 (UK) [24]	11 10/11 isolates were fluconazole-resistant	25 to 64 years old Patients had symptoms such as itching, soreness, and discharge persisting for at least three months, with a median duration of one year. Fluconazole resistance determined via yeast cultures with susceptibility testing, as per CLSI standards.	*C. albicans* *C. glabrata* *S. cerevisiae* *C. krusei* *C. lambica* *C. dubliniensis*	Oral voriconazole, with or without concomitant topical agents	**Voriconazole**: 400 mg twice on day 1, and then 200 mg twice every 24 h for 13 days. **Nystatin Pessaries**: 100,000 IU intravaginally, typically used for 12 to 14 nights. **Miconazole Cream**: 2% cream, applied intravaginally (5 g per night) for 14 nights. **Boric Acid Pessaries**: 600 mg intravaginally, used daily for 14 nights.	Retrospective Case Report	All cases were treated with oral voriconazole. 6/11 also used concomitant topical agents. Symptom reduction and yeast clearance in 8/11. 2/11 initially had a partial response but achieved resolution after a second course of voriconazole
Fan SR and Liu XP, 2011 (China) [20]	283	Resistance determined using the E-test method following the CLSI guidelines.	*C. albicans* *C. glabrata* *S. cerevisiae* *C. tropicalis* *C. famata* *C. parapsilosis* *C. krusei*	**Nystatin Vaginal Tablets** **Oral Fluconazole**	**Nystatin Group**: 20 MU/day of vaginal nystatin for 14 days. **Fluconazole Group**: 150 mg in two doses, and the second dose administered 3 days after the first.	Non-RCT	In vitro susceptibility to nystatin by all *Candida* species.
Richter SS et al., 2005 (USA) [22]	429	Resistance determined using a broth microdilution method as per NCCLS guidelines. MIC values used to classify the resistance.	*C. albicans* *C. glabrata* *C. parapsilosis* *C. krusei* *S. cerevisiae* *C. tropicalis* *C. lusitaniae* *Trichosporon* sp.	Fluconazole Voriconazole Caspofungin Micafungin Flucytosine Miconazole Clotrimazole Ketoconazole	Treatment with fluconazole was 200 mg orally every other day for three doses. *C. glabrata* were treated with boric acid (600 mg intravaginally once daily for 2 weeks).	Retrospective RWE	***Candida albicans***: **Fluconazole**: 61.5% reported improvement **Clotrimazole**: Clinical improvement in 57.1% of episodes treated. **Econazole**: Clinical improvement in 40% of episodes treated. ***Candida glabrata***: **Boric Acid**: Improvement in 48.6% of episodes treated. **Clotrimazole**: Clinical improvement in 57.1% of episodes treated. **Econazole**: Clinical improvement in 40% of episodes treated. ***Candida krusei***: **Fluconazole**: Two episodes showed symptomatic improvement despite high MIC values (32 and 128 µg/mL).
Singh S et al., 2002 (USA) [35]	12 women aged between 32 to 63 years, with a mean age of 44 years.	Resistance determined using the broth microdilution method as per NCCLS guidelines. MIC for fluconazole was very high, ranging from 32 to 164 µg/mL, indicating significant resistance.	*C. krusei* (primary focus) *C. albicans* *C. tropicalis* *C. glabrata* *C. guilliermondii*	**Oral**: Ketoconazole Itraconazole **Topical**: Boric Acid Clotrimazole Amphotericin B Nystatin Flucytosine	**Boric Acid**: 4 to 6 weeks of topical treatment, 600 mg intravaginally. **Clotrimazole**: 6 to 36 weeks of topical treatment, with varying dosages depending on the patient’s response. Ketoconazole: 10 days of oral therapy. **Nystatin**: 4 weeks of topical treatment. **Amphotericin B**: 2 weeks of 3% topical cream. **Itraconazole**: Not specified duration but failed to respond to therapy. **Flucytosine**: 2 weeks of topical treatment, followed by 4 weeks of nystatin.	Retrospective RWE	Cure was achieved in 4 of the 6 patients treated with boric acid. Clotrimazole also showed success in 2 out of 3 patients. However, several cases were refractory to multiple treatments, indicating the challenging nature of treating *C. krusei* vaginitis
Nyirjesy P et al., 2022 (USA) [34]	18 years or older Symptomatic moderate-to-severe acute VVC. VSS score of ≥7, a positive microscopic examination with 10% potassium hydroxide revealing yeast forms, and a vaginal pH of ≤4.5.	Resistance was measured using in vitro susceptibility testing as per CLSI guidelines	*C. albicans* *C. glabrata* *C. krusei* *C. auris* *C. parapsilosis* *C. tropicalis*	Ibrexafungerp: Oral Fluconazole: Oral	Ibrexafungerp: 300 mg twice for 24 h Fluconazole: 150 mg for 24 h.	RCT	Clinical cure rates were 51.9% for ibrexafungerp versus 58.3% for fluconazole (day 10). 70.4% for ibrexafungerp versus 50.0% for fluconazole (day 25). The need for rescue medication was lower with ibrexafungerp (3.7%) compared to fluconazole (29.2%).
Kalkan Ü et al., 2021 (Turkey) [36]	18 to 50 years with recurrent VVC	Inclusion required at least one episode verified by microscopy Resistance was not directly measured as focused on boric acid as an alternative treatment	*C. albicans* *C. glabrata*	**Boric Acid**: Intravaginal **Vaginal Estriol-Lactobacilli Combination**: Intravaginal	**Boric Acid**: 600 mg intravaginal suppositories daily for 14 nights during induction, followed by maintenance therapy of 600 mg for another 5 days, starting on the first day after menstruation. Dose adjusted to 300 mg every 24 h instead if irritation occurred. **Estriol-Lactobacilli**: Administered after boric acid treatment, containing 0.03 mg estriol and live *Lactobacillus acidophilus*.	Retrospective RWE	The overall success rate of boric acid-based therapy at the one-year follow-up was 94.8%.
D. Ray et al., 2007 (India) [40]	112 female diabetic patients 77 patients had Type 2 diabetes, and 35 had Type 1 diabetes 40.2–41.2 years of age	Natural resistance of *Candida glabrata* to fluconazole, particularly in diabetic women with VVC	*C. glabrata*: 68 patients (61.3%) *C. albicans*: 32 patients (28.8%) *C. tropicalis*: 4 patients (3.6%)	Fluconazole Boric acid	Single-dose oral 150 mg of fluconazole. Boric acid vaginal suppositories at 600 mg per day for 14 days.	RCT	**Mycological Cure Rates**: ***C. glabrata***: Boric acid: 72.4% cure rate (21 of 29 patients). Fluconazole: 33.3% cure rate (10 of 30 patients). ***C. albicans***: Boric acid: 61.1% cure rate (11 of 18 patients). Fluconazole: 85.7% cure rate (12 of 14 patients).
M.A. Kennedy and J.D. Sobel, 2010 (USA) [37]	120 women with NAC isolated 44.7 mean years of age	Resistance inferred from clinical failures in the treatment of NAC species, especially *C. glabrata*, which showed persistence despite fluconazole	*C. glabrata*: 80 patients. *C. parapsilosis*: 32 patients. *C. lusitaniae*: 8 patients	Boric Acid Fluconazole	**For *C. glabrata***: Boric acid vaginal capsules (600 mg) used nightly for 14 to 21 days. **For *C. parapsilosis***: Fluconazole primary treatment, with dosing similar to that used for *C. albicans*. ***C. lusitaniae*** was treated with either boric acid or fluconazole.	Retrospective RWE	*Candida glabrata*: Overall, 35% (13/37) of treated patients had both clinical and mycological resolution *Candida parapsilosis*: 45% (9/20) had complete resolution of symptoms and eradication of yeast. *Candida lusitaniae*: 42.9% (3/7) had complete resolution of symptoms and eradication of yeast. Boric acid is more effective for treating *C. glabrata*, particularly in cases of azole resistance, with 35% achieving full resolution, whereas fluconazole has a high failure rate for this species.
Nyirjesy P et al., 2005 (USA) [39]	51 women with chronic VVC complaints who had positive cultures for *Candida parapsilosis* 19–86 years of age	NAC species, including *C. parapsilosis*, may have higher MICs for azoles but did not directly measure resistance	*C. parapsilosis* *C. albicans* *C. glabrata* *C. lusitaniae*	Boric acid Buconazole Fluconazole Miconazole	**Boric Acid**: 600 mg twice daily for 2 weeks. **Fluconazole**: 200 mg twice weekly for 1 month. **Buconazole**: Two vaginal applicators, 1 week apart. **Miconazole**: One vaginal applicator, nightly for 7 days.	Retrospective RWE	Mycological Cure: Fluconazole: 17 out of 19 cases (89.5%). Buconazole: 7 out of 7 cases (100%). Boric acid: 6 out of 6 cases (100%). Miconazole: 1 out of 1 case
M.G. Martens et al., 2022 (USA) [38]	219 patients Mean age 35 years	Fluconazole resistance was assessed through MIC	*C. albicans* (76.1%) *C. glabrata* (11.8%) *C. parapsilosis* (5.4%) *C. tropicalis* (4.1%)	Oteseconazole Fluconazole	**Oteseconazole** Induction phase: 600 mg (4 × 150 mg capsules) on day 1 and 450 mg (3 × 150 mg capsules) on day 2. Maintenance phase: 150 mg once weekly for 11 weeks. **Fluconazole** Induction phase: 150 mg on day 1, day 4, and day 7 (3 doses total). Maintenance phase: Given a placebo for the same duration.	RCT	Oteseconazole 5.1% of participants had a recurrent VVC episode Fluconazole/placebo: 42.2% of participants had a recurrent VVC episode
Ratner JC et al., 2024 (UK) [33]	5461 adult patients 3-year period (April 2018–March 2021)	Fluconazole resistance assessed using the disc diffusion method and Sensititre YeastOne assay as per CLSI	*C. albicans* (>85% of isolates; declining over the years). NAC: *Nakaseomyces glabrata [Candida glabrata],* *Pichia kudriavzevii [Candida krusei]*, *C. dubliniensis*, *Meyerozyma guilliermondii, Clavispora lusitaniae,* *C. parapsilosis,* *C. tropicalis.*	Fluconazole Itraconazole Voriconazole Clotrimazole Amphotericin B	**Fluconazole**: Induction dose of 150 mg orally three times per week, followed by 150 mg once weekly for 6 months **Clotrimazole**: A 500 mg pessary (vaginal tablet) as single-dose treatment. Recurrent cases: 100 mg pessaries daily for 6 days. **Amphotericin B/Voriconazole/Itraconazole**: Dosage N/A	Retrospective RWE	Patients with fluconazole-resistant (both *Candida albicans* and NAC) faced higher treatment failure, necessitating multiple courses of alternative antifungals. NAC species were generally more resistant to standard treatments, leading to a lower success rate when fluconazole was used

VVC: vulvovaginal candidiasis; CLSI: Clinical and Laboratory Standards Institute; MIC: minimum inhibitory concentration; NCCLS: National Committee for Clinical Laboratory Standards; RCT: randomized controlled trial; RWE: Real World Evidence.

## Data Availability

No new data were created or analysed in this study. Data sharing is not applicable to this article.
